# A novel homozygous variant of *GPR98* causes usher syndrome type IIC in a consanguineous Chinese family by next generation sequencing

**DOI:** 10.1186/s12881-018-0602-0

**Published:** 2018-06-11

**Authors:** Chunli Wei, Lisha Yang, Jingliang Cheng, Saber Imani, Shangyi Fu, Hongbin Lv, Yumei Li, Rui Chen, Elaine Lai-Han Leung, Junjiang Fu

**Affiliations:** 1 State Key Laboratory of Quality Research in Chinese Medicine, Macau Institute For Applied Research in Medicine and Health, Macau University of Science and Technology, Taipa, Macao, Special Administrative Region of China; 2 Key Laboratory of Epigenetics and Oncology, Research Center for Preclinical Medicine, Southwest Medical University, Luzhou, Sichuan China; 3 Chemical Injuries Research Center, Baqiyatallah University of Medical Sciences, Tehran, Iran; 4 The Honors College, University of Houston, Houston, TX USA; 5 Department of Molecular and Human Genetics, Baylor College of Medicine, Houston, TX 77030 USA; 6 Department of Ophthalmology, Affiliated Hospital of Southwest Medical University, Luzhou, Sichuan China; 7 Guangzhou Institute of Respiratory Disease, State Key Laboratory of Respiratory Disease, The 1st Affiliated Hospital of Guangzhou Medical College, Guangzhou, China; 8 Respiratoire Medicine Department, Taihe Hospital, Hubei University of Medicine, Hubei, China

**Keywords:** Usher syndrome type IIC, GPR98, Nonsense mutation, Molecular diagnosis, Next generation sequencing, Consanguineous marriage

## Abstract

**Background:**

Usher syndrome (USH) is a common heterogeneous retinopathy and a hearing loss (HL) syndrome. However, the gene causing Usher syndrome type IIC (USH2C) in a consanguineous Chinese pedigree is unknown.

**Methods:**

We performed targeted next-generation sequencing analysis and Sanger sequencing to explore the *GPR98* mutations in a USH2C pedigree that included a 32-year-old male patient from a consanguineous marriage family. Western blot verified the nonsense mutation.

**Results:**

To identify disease-causing gene variants in a consanguineous Chinese pedigree with USH2C, DNA from proband was analyzed using targeted next generation sequencing (NGS). The patient was clinically documented as a possible USH2 by a comprehensive auditory and ophthalmology evaluation. We succeeded in identifying the deleterious, novel, and homologous variant, c.6912dupG (p.Leu2305Valfs*4), in the *GPR98* gene (NM_032119.3) that contributes to the progression of USH2C. Variant detected by targeted NGS was then confirmed and co-segregation was conducted by direct Sanger sequencing. Western blot verified losing almost two-thirds of its amino acid residues, including partial Calx-beta, whole EPTP and 7TM-GPCRs at the C-terminus of GPR98. Furthermore, our results highlighted that this p.Leu2305Valfs*4 variant is most likely pathogenic due to a large deletion at the seven-transmembrane G protein-coupled receptors (7TM-GPCRs) domain in GPR98 protein, leading to significantly decreased functionality and complex stability.

**Conclusions:**

These findings characterized the novel disease causativeness variant in *GPR98* and broaden mutation spectrums, which could predict the pathogenic progression of patient with USH2C, guide diagnosis and treatment of this disease; and provide genetic counseling and family planning for consanguineous marriage pedigree in developing countries, including China.

## Background

Usher syndrome (USH) is a common heterogeneous retinopathy and a hearing loss (HL) syndrome; the disease is also known as Usher-Hallgren syndrome, Hallgren syndrome, or retinitis pigmentosa (RP)-dysacusis syndrome [1, 2]. It has high prevalence, often estimated to be roughly 1 in 6000 individuals and 1 in 6 people with RP, covering nearly 50% of all deaf-blindness cases [3, 4]. USH is mostly inherited in an autosomal recessive pattern and its clinical subtypes can be determined by the presence of arreflexia of sensorineural HL: USH1, USH2, and USH3 [2, 5].

Pathogenically, Usher syndrome is mapped by at least eleven *USH* genes which contribute to inheritance in USH1: *MYO7A, USH1C, CDH23, PCDH15, USH1G, USH2A, GPR98, DFNB31, CLRN1*, and *PDZD7* [6–11]. Usher syndrome type II is the most common form of USH syndrome, leading to severe RP in the second decade of life along with congenital, moderate-to-severe HL. To date, USH2 loci are mutagenetically heterogeneous in three different genes: *USH2A* (USH2A, OMIM 276901) [12], *GPR98* (USH2C, OMIM 605472) [8, 13], and *DFNB31* (USH2D, OMIM 611383) [14]. *PDZD7* is a modifier gene found in USH2 patients [15]. To our knowledge, *USH2A* is the most prevalent mutated gene in USH2 patients, consisting of an estimated 80% of all patients inflicted with USH2, whereas *GPR98* mutations account for a small but significant number of inflicted patients [16].

The human G protein-coupled receptor 98 (*GPR98*) gene (NM_032119.3), located at chromosome 5q14.3, is also aliased as *FEB4*, *USH2B*, *USH2C*, *VLGR1*, *VLGR1b*, *ADGRV1* and *MASS1*. The protein, encoded by *GPR98* gene, is a part of an Usher interactome as G protein-coupled receptors (GPCRs). Functionally, GPCRs, as a seven-transmembrane domain receptors (7TM receptors) (7TM-GPCRs), are integral membrane proteins that possess seven membrane-spanning domains or transmembrane helices [17]. In USH2C patients, GPCRs are critical for the proper hair cell development and maintenance of photoreceptors structures, in which it may allow connecting cilium to anchor the inner segment of the photoreceptors [18]. As a seven-transmembrane receptor in the retinal cells, the extracellular loops of GPCRs also contain two highly conserved cysteine residues that form disulfide bonds to stabilize the receptor structure [19, 20]. Even with emerging evidence, GPCR’s function in the USH2 disease is not well described yet.

Although USH2C has been studied in regard to mutational screening, characterization of different variants in the whole region of *GPR98* gene, is still unknown. Clinically and genetically, this is a necessary issue to be addressed as the USH2C patients who desperately need genetic counseling have no definitive answer [15, 19].

Next generation sequencing (NGS) is one of the newest, most cost-effective molecular analysis technique mainly used to screen pathogenic variants in inherited heterogeneous disorders [21–23]. In this study, we aimed to categorize pathogenic and disease-causing variants by targeted NGS technologies-based mutation screening in a Chinese USH2C pedigree with consanguineous marriage. Our results successfully found a deleterious, novel, and pathogenic mutation c.6912dupG (p.Leu2305Valfs*4), in the *GPR98* gene. This less commonly mutated gene could possibly predict the progression of USH2C, contributing to the progression of causativeness and susceptibility in USH2C patient. This study provides detailed information that will help find the genetic and clinical data to support the contention that the autosomal recessive mutation in *GPR98* is most likely pathogenic by damaging the GPR98 protein structure and protein/protein interaction.

## Methods

### Patient recruitments

**Fig. 1:**
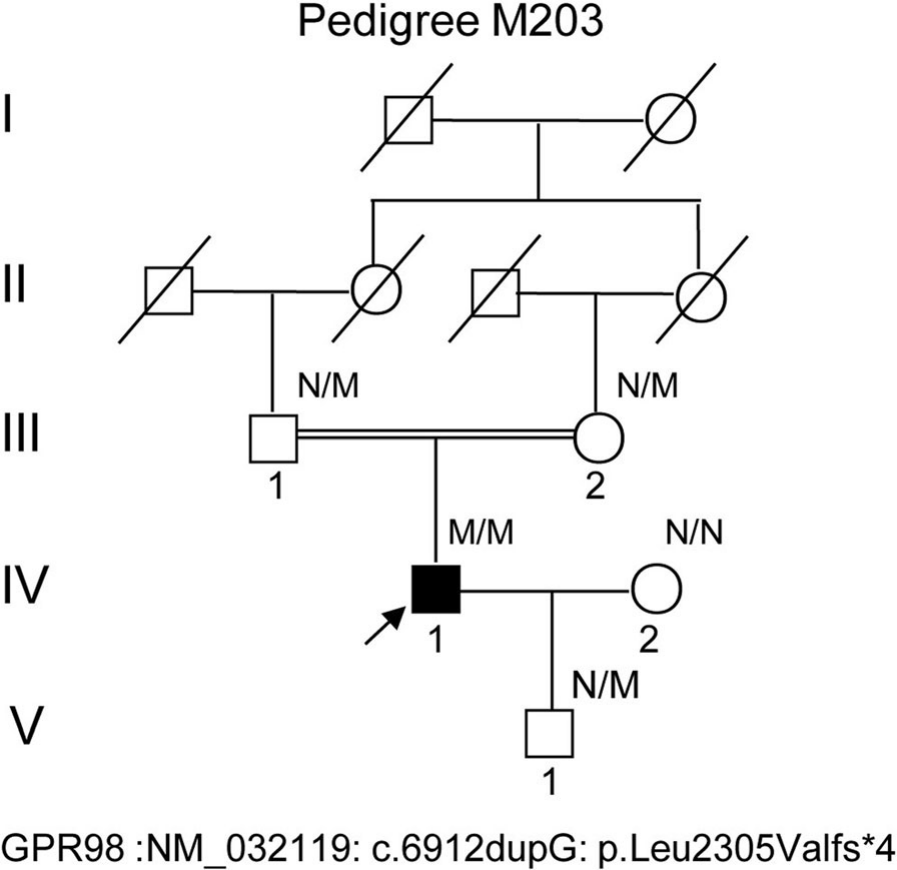
The pedigree for USH2C in Chinese with consanguineous marriage. Family numbers and disease-causing variant is noted above pedigree. Normal individuals are shown as clear circles (females) and squares (males), whereas affected individual is shown as a filled symbol. III:1 and III:2 are consanguineous marriage with symbol “**=**”. The patient above the arrow indicates the proband (IV:1). The arrow indicates the patient by next generation sequencing with G duplication mutation of *GPR98* gene NM_032119.3: c.6912dupG: p.Leu2305Valfs*4. “M” indicates the mutant allele of GPR98, whereas “N” indicates normal allele without mutation

This research was approved by the Ethical Committees of the Southwest Medical University. All written informed consents and procedures adhered to the tenets of the Declaration of Helsinki (1983 Revision). A written informed consent to genetic testing was obtained from all enrolled subjects according to the recommendations of the local ethics committee of Southwest Medical University before inclusion into the study. A pedigree for a consanguineous marriage, five-generations, eleven-Chinese members including one patient proband with both hearing loss and vision defect, was enrolled (Fig. 1, pedigree IV: 1, arrow, molecular No. M203). A complete historic interview enabled us to draw an extended pedigree from the family and to confirm the inheritance pattern. The proband has been clinically evaluated according to the criteria recommended by the USH Consortium [20]. A comprehensive auditory and ophthalmology evaluation was performed upon the proband, including the followings: otoscope examination, tympanometry, best corrected visual acuity (BCVA) measurements, slit-lamp biomicroscopy and color vision, fundus photography, visual field tests. The retinal phenotypes, thickness, and structure change of proband were examined by full field electroretinograms and optical coherence tomography instruments (OCT, Carl Zeiss, Germany), according to the standard protocol. Audiograms were measured by pure-tone audiometry at different frequencies of 0.25, 0.5, 1, 2, 4, and 8 kHz in the proband.

### DNA sampling

Whole blood samples from all members of the M203 pedigree were collected (Fig. 1). Human genomic DNAs (gDNA) from peripheral leukocytes were extracted using the previously described phenol/chloroform method [24, 25]. The concentration of the extracted gDNAs was measured by a NanoDrop2000 spectrophotometer (Thermo Scientific, Wilmington, DE, USA). DNAs from 100 ethnically matched Chinese individuals without any hearing and retinal diseases were recruited as normal controls.

### Targeted NGS and data analysis

Sequencing analyses were performed as described using the capture Agilent probes that was used in previously published studies [22, 26, 27]. The design of targeted capture panels with 195 genes has been described in previous literature, according to the Illumina paired-end libraries [22, 26, 28]. Variant filtering and homozygosity mapping were performed using a paired-end sequencing Illumina, which was aligned to the human hg19 reference genome using Burrows-Wheeler Aligner version 0.6.1 and available public online UCSC database (http://genome.ucsc.edu/) [29], as previously described in detail [22, 26]. The data from targeted NGS were analyzed as described [21, 27, 30]. The functional classification of proteins via subfamily domain architectures for GPR98 was performed through an online system (https://www.ncbi.nlm.nih.gov/Structure/cdd/wrpsb.cgi) [30].

### Variant verification and segregation analysis

The locus-specific primers for variant verification and segregation analysis were designed using online Primer3 program (website: http://bioinfo.ut.ee/primer3-0.4.0/) (left primer 5’-GGGACACTCGGCAATGTTAC-3′; right primer 5′- CCAAAATGTTGAACAATGCAA -3′). The mutant position should locate at least 50 bps away from the 3′-end of both left and right primers. A product with 422 bps was amplified by PCR using gDNA as the template [25]. Sanger sequencing was conducted to validate the suspected pathogenic variants and segregation analysis using 3500DX ABI Genetic Analyzer (Foster City, USA).

### Western blot analysis

One ml of blood from the affected proband with mutant homozygous type (pedigree IV:1, Fig. 1); one ml of blood from the mutant heterozygous type (M204) (pedigree III:1, Fig. 1); and one ml of blood from the wild type individual in this Chinese family (pedigree IV:2, Fig. 1) were collected, respectively, to isolate the leukomonocytes from the red cells using lymphocyte separation medium (TBD, China), and then lysed using the EBC lysis buffer [31, 32]. The concentration of the proteins was determined with protein quantification kit-Rapid (Sigma, USA). Then, lysates were separated onto a 7% SDS-polyacrylamide gel and transferred to a PVDF membrane (Millipore, Corporation, Billerica, MA, USA). The membrane was blocked with 5% milk without fat in 1 × TBST buffer (1 × TBS plus 0.05% Tween-20) for 2 h at room temperature. Primary GPR98 antibody (Cat #: abs139741a, Absin Bioscience Co., Ltd., Shanghai, China) with 1:1000 dilution and tubulin antibody (Cat #: T0198, Sigma, USA) with 1:2500 dilution were incubated respectively at 4 °C for 13 h with gentle shaking. After the membranes were washed three times with 1 × TBST buffer for 15 min per wash, the secondary antibodies tagged with horseradish peroxidase (HRP) (1:2000 dilution) were added to the membranes and incubated at room temperature for 2 h with gentle shaking. After another three washes with 1 × TBST buffer for 10 min per wash, protein bands were recorded by the digital imaging system (Universal Hood II, Bio-Rad Lab, Italy) [32].

## Results

### Phenotype for proband with usher syndrome

**Fig. 2:**
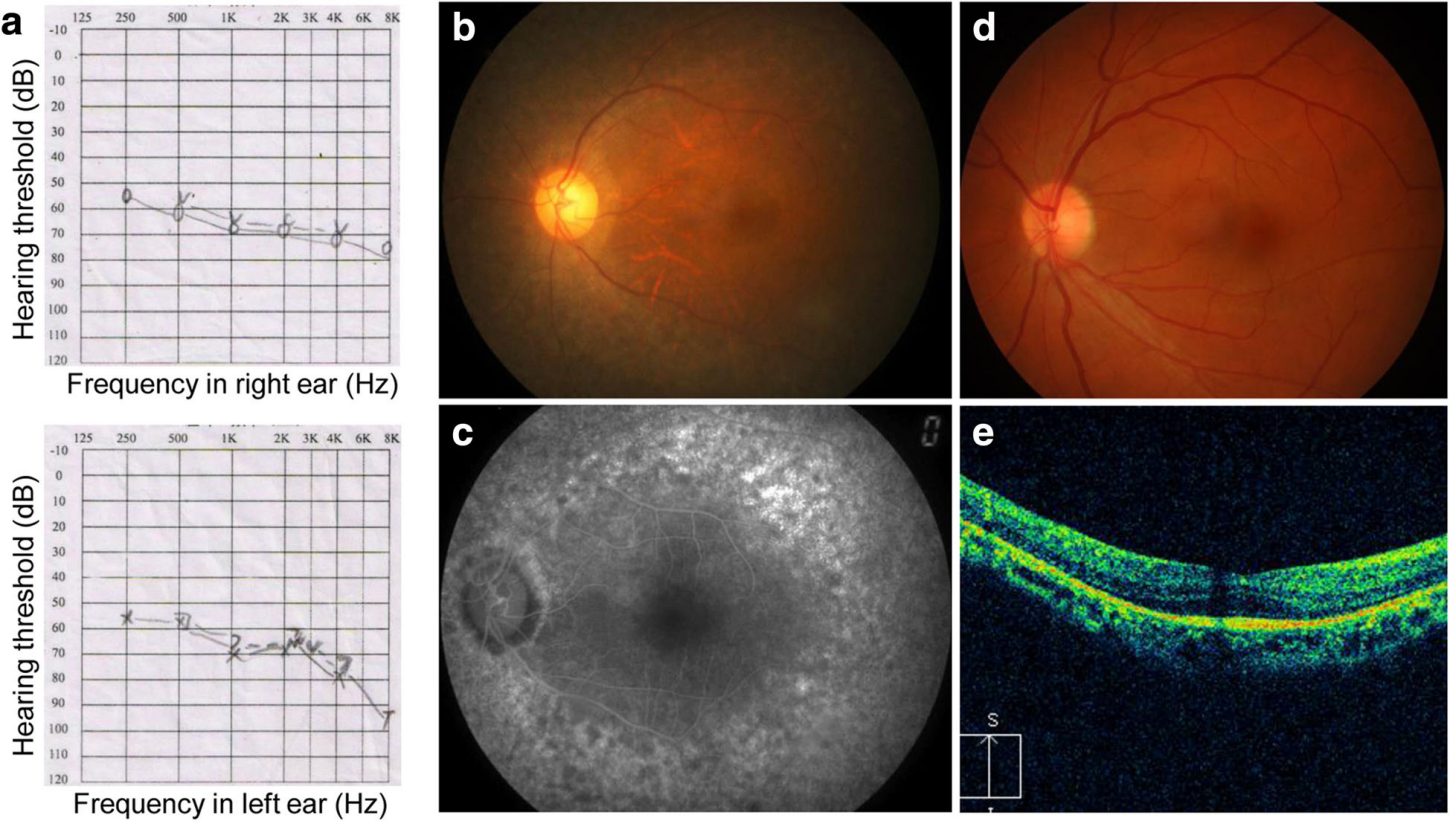
Ear audiograms and retinal phenotypes of proband IV: 1. **a**. Physiologic audiograms of both ears of proband IV:1. The audiograms reveal bilateral slightly down-sloping and show a moderate-to-severe hearing loss across all frequencies. Representative fundus photographs of patient (**b, c**) and normal control (**d**). The proband clearly shown the “salt and pepper” pigment mottling pattern, severe RPE atrophic changes and the transparent in the macula. The vessels are very thin. Optical coherence tomography in the right eye (**e**)

The pedigree with consanguineous marriage was recruited from Department of Ophthalmology in Southwest Medical University (Fig. 1) when the proband was 32-year-old. The father of proband claimed his son was found to have hearing defects when he was one and half year old during otitis media therapy at the local county hospital, and had a gradual decrease of vision, which was revealed when he turned 20-year-old. Pure tone audiometry testing presented prelingual deafness with bilateral slightly down-sloping and showed a moderate-to-severe sensorineural hearing loss across all frequencies (Fig. 2a). The retinal phenotypes of the proband were compared to the normal control (Fig. 2b-e). The presenting symptoms of proband were shown as decreased central vision and visual acuity, and prominent presence of fundus flecks in the posterior pole of the retina. The FA (fluorescein angiogram) results (pedigree IV:1, Fig. 1) showed a “salt and pepper” pigment mottling pattern; severe RPE atrophic changes and the transparency of the macula; and fundus abnormalities (Fig. 2b-c). Optical coherence tomography (OCT) showed that marked thinning and disruption of the photoreceptor layer, choroid and the retinitis pigment epithelium (Fig. 2e). There was no history of imbalance gait or vertigo. Thus, it was determined that the patient had likely presence of USH2 (Fig. 1). Family history has shown no other members with hearing losses or visual problems.

### Mutation identification for the *GPR98* gene

**Fig. 3:**
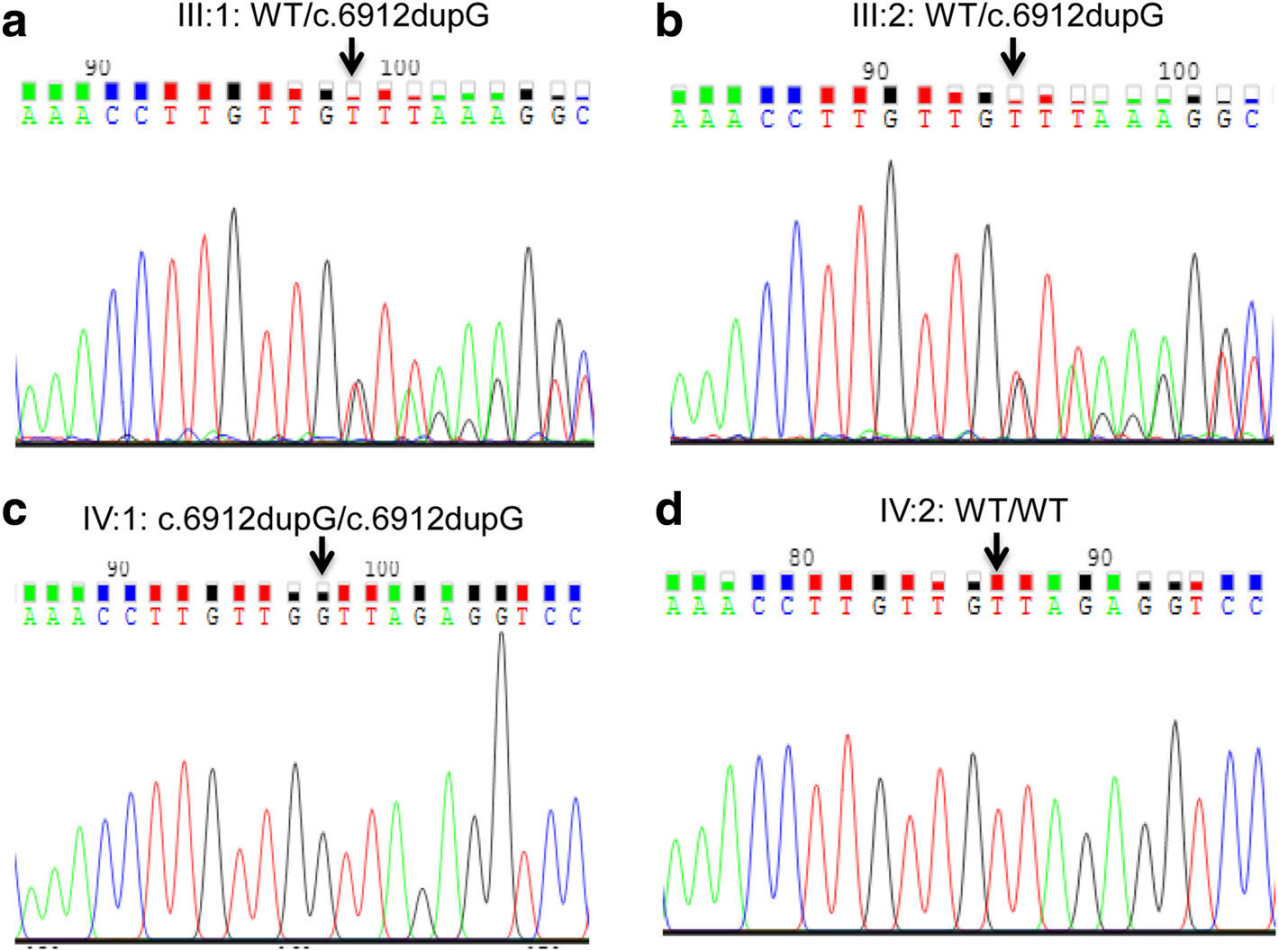
Validation and segregation analysis by Sanger sequencing. **a, b**, **c** and **d** indicate the sequencing results in III: 1 (M204, heterozygous type), III: 2 (heterozygous type), IV: 1 (M203, mutant homozygous type) and IV:2 (wild type, normal control: no eye and ear disease history in her family), respectively. The arrows indicate the duplication at the nucleotide position for *GPR98* gene NM_032119.3: c.6912dupG: p.Leu2305Valfs*4

The proband was analyzed using target capture panel sequencing. This panel covers coding exons and flanking splicing junctions for 195 known retinal disease genes, including *GPR98* [22, 28]. High quality next-generation sequencing data were obtained. The NGS analysis in the proband identified homozygous variant c.6912dupG for the *GPR98* gene (NM_032119.3). We successfully identified the duplication mutation was c. 6912dupG in exon 31, resulting in a missense change of Leucine at position 2305 to Valine and frameshift mutation with termination codon at position 2310 on GPR98 protein (p.Leu2305Valfs*4). After checking this variant on the exome variant server and ExAC databases showed this variation is novel. This mutation has not been reported in single nucleotide polymorphisms, and is perfectly co-segregated with the USH2 phenotype shown by Sanger sequencing. The c.6912dupG variant was not found in 100 unrelated, ethnically matched normal controls. The representative results of the Sanger sequencing of this variant are shown in Fig. 3. As we estimated, the consanguineous proband’s parents (Fig. 3a&b, pedigree: III: 1&2) were heterozygous for c.6912dupG. The proband was homozygous for the mutation of *GPR98* (Fig. 3c), whereas his wife (pedigree IV: 2, Fig. 1) was homozygous for the wild type allele of *GPR98* (Fig. 3d). The proband wife genotype is not relevant since there is no consanguinity. This confirmation results showed comprehensive co-segregation in this pedigree family association furthering population studies and pinpointing the roles in USH2C pathogenesis.

### Change of GPR98 protein

**Fig. 4:**
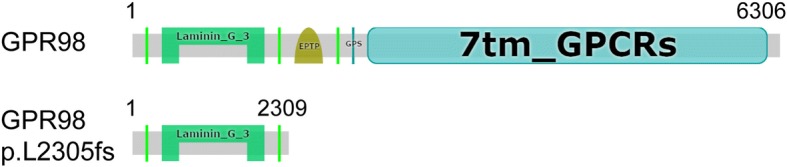
The conserved domains analysis for GPR98 amino acid residues wild type and its mutant protein c.6912dupG: p.Leu2305Valfs*4. The conserved domains analysis was performed through the online system (https://www.ncbi.nlm.nih.gov/Structure/cdd/wrpsb.cgi). Wild type protein contains domains Laminin-G-3, EPTP, Calx-beta (green box), and 7TM-GPCRs, whereas mutant protein only contains domains Laminin-G-3, and Calx-beta, which loses almost two-thirds of amino acid residues including partial Calx-beta, and whole EPTP and 7TM-GPCRs. Laminin-G-3: Laminin G domain; 7TM-GPCRs: seven-transmembrane (7TM) G protein-coupled receptors (GPCRs); GPS: GPCR proteolysis site (motif). “p.L2305 fs” indicates “p.Leu2305Valfs*4”

We recognized a single nucleotide homozygous duplication (c.6912dupG) of *GPR98* gene in this USH2C family, leading to a missense change of Leucine at position 2305 to Valine and a frameshift mutation in the reading frame at amino acid position 2305 and another 4 incorrect amino acids (RGPG) after codon 2305, followed by premature termination at codon 2310 (p.Leu2305Valfs*4), with a total of 2309 amino acids of mutant GPR98 protein (Fig. 4). Conserved domains analysis for GPR98 protein amino acid residues of the wild type and the mutant c.6912dupG: p.Leu2305Valfs*4 revealed that the wild type protein contains domains Laminin-G-3, EPTP, Calx-beta (green box), and 7TM-GPCRs, whereas the mutant protein only contains domains Laminin-G-3, and Calx-beta, losing almost two-thirds of its amino acid residues, including partial Calx-beta, whole EPTP and 7TM-GPCRs at the C-terminus of GPR98, which is considered as the cause of the disease. Notable, EPTP domain is a common protein interaction domain links identified epilepsy gene *Epitempin*.

**Fig. 5:**
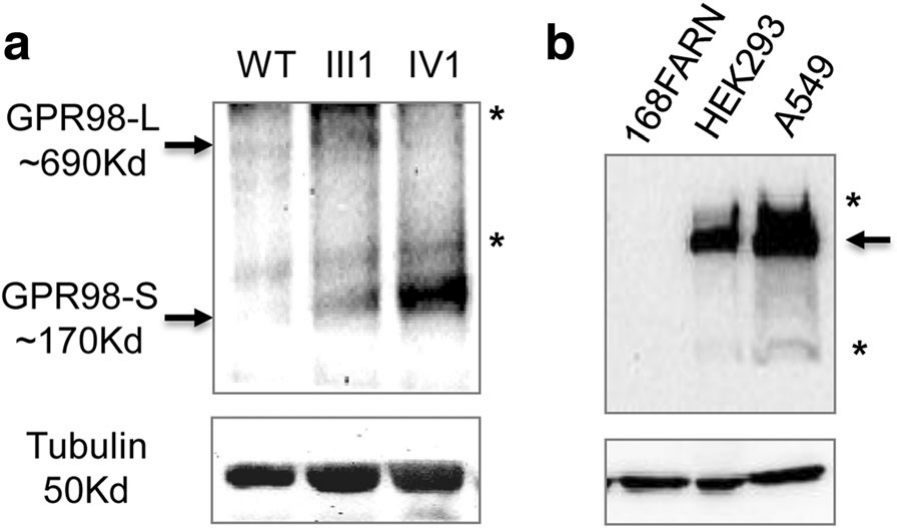
Western blot results of GPR98. **a**. Western blot results for samples III: 1 (M204), IV: 1 (M203, mutant homozygous type) and IV: 2 (wild type, normal control), respectively. **b**. Western blot results for cancer cell lines. The arrows indicate the large size (GPR98-L) and/or small size (GPR98-S) forms of GPR98 with p.Leu2305Valfs*4. “*” in the image indicates non-specific bands, respectively

To verify the predicted change in GPR98 protein, the GPR98 in proband (pedigree IV:1, Fig. 1) was compared to the heterozygous type (pedigree III:1, Fig. 1), the wild type, and the normal control (pedigree IV:2, Fig. 1), respectively, by western blot analysis. The results are shown in Fig. 5a; It is worthy to note that a smaller band (GPR98-S; ~ 170 KD) was presented only in mutant homozygous type of USH2C patient (pedigree IV:1, Fig. 5a, lane “IV1”) and a larger (GPR98-L; ~ 690 KD) band was presented only in wild type (normal control) (pedigree IV:2, Fig. 5a, lane “WT”) in GPR98 protein, respectively. This smaller band is the probable, truncated GPR98 protein due to the frameshift mutation (p.Leu2305Valfs*4). In the heterozygous type of USH2C carrier III: 1, we identified both a smaller band (GPR98-S; ~ 170 KD) and a larger band (GPR98-L; ~ 690 KD) (Fig. 5a, lane “III1”). We also noticed that the signals of smaller band GPR98-S in proband (IV:1) with homozygous mutation are stronger than that of carrier (III:1) with heterozygous mutation due to copy number difference of mutant *GPR98* gene. To make sure whether SDS-PAGE of 7% acrylamide can used to detect a large protein of ~ 700 KD successfully, different cancer cell lines were used for WB, which do show specific band with GPR98 (Fig. 5b and data not shown). These findings suggest that the c.6912dupG: p.Leu2305Valfs*4 variant in the USH2C patient could affect the GPR98 protein function, by causing a premature frameshift in the structure.

## Discussion

By identifying *GPR98* mutation, the current study retrospectively performed targeted NGS on USH Chinese family with consanguineous marriage to identify a disease-causing variant responsible for the USH2C syndrome (Usher Syndromes type IIC) which thereafter characterized at the molecular level. USH2C is usually characterized by moderate-to-severe hearing loss, latter onset of retinal degeneration and normal vestibular function [2, 8, 33]. For the first time, our findings introduced this family with a novel c.6912dupG: p.Leu2305Valfs*4 *GPR98* frameshift mutations, which lead to protein truncation. These findings highlighted that this novel variant in the GPR98 is likely the deleterious variant, thereby expanding the *GPR98* mutation spectrums for USH2C syndrome.

Clinical subtyping of USH hinders the critical therapy step, which allows early molecular diagnosis of patient’s families through genotype-phenotype descriptions and characterization of any new disease-causing gene variants in the molecular laboratory [16, 20]. Following this approach, USH mutation genes were documented and clinically used for subtypes of Usher or other retinal diseases. *GPR98* mutations account for a small portion for mutation that causes USH2C. Till now, ten deleterious mutations, including small insertions, point mutations, deletions, and splicing alterations were identified in *GPR98* [34]. This patient is successfully characterized as USH2C syndrome by combination of *GPR98* mutation identification, hearing loss observation, the retinal pigment epithelium abnormality and rod-cone degeneration [11].

It is well known that NGS is one of the newest, most cost-effective molecular analysis technique mainly used to identify pathogenic variants in inherited heterogeneous disorders [21, 22, 27]. Literature reviews show NGS is a predominantly accurate technique which adds to previous differential imaging techniques that have been able to characterize and map more genetically heterogeneous macular degeneration/dystrophy (MD) disorder, such as RP, STGD-like MD, LCA, and USH [28, 35, 36]. To address this issue, according to NGS-based models, we have succeeded in identifying a new *GPR98* disease-causing variant in a Chinese USH2C family (c.6912dupG: p.Leu2305Valfs*4).

The encoded GPR98 protein (NP_115495.3) is a very large protein found in humans, with a size of 6306 amino acids and consisting of 90 exons (NM_032119.3, largest isoform b with predicted 605KD). Structurally, it belongs to the subfamily of the GPCR2 family for G-protein signaling or large N-terminal family B (LNB) of 7TM receptors (LN-TM7 subfamily) [37]. Specifically, this protein, which has several domains, is detected to be ~ 690 KD in molecular mass by western blot (Fig. 5), involved in the signaling pathways of GPCRs in the inner ear and retinal cells, and possibly leads to variable ratios of mutant/normal transcripts in these cells [3, 38]. Its function in the retinal cells guaranteed photoreceptor cell maintenance and visual perception of the retinal [20]. Similarly, the GPR98 protein may localize in specific subcellular compartments in spiral ganglion cells of the cochlea that assist in maintaining stereociliary cohesion [3]. The mutation’s gene expression may affect the cochlear stereociliary bundle and cause hearing loss [38, 39].

In last decade, homozygous mutations in the *GPR98* gene were reported in a consanguineous Tunisian family [40], Iranian family [8], German family [41], and French family [41], all of which are linked to USH2C syndrome phenotype. These genotype/phenotype correlations are helpful in the verification of GPR98 function in the USH2C syndrome [42]. Most of mutations are located in the 7TM-GPCRs functional domain in the *GPR98* gene. All predicted premature terminations have led to the dysfunction of the GPR98 complex, suggesting that this protein participates in cell adhesion and signal transduction of sensory retinal cells. In this study, we first time report a novel, large deletion of the 7TM-GPCRs of GPR98 protein in a large consanguineous Chinese USH2C family. Conserved domain analysis for GPR98 protein shows this region of GPR98 is highly conserved and predicts a globular domain that is involved in retinal cell-cell signaling pathways [20, 30]. The frameshift of deleterious c.6912dupG: p.Leu2305Valfs*4 variant might distribute protein abnormal location and affect normal protein/protein interactions after deleting the functional domain of GPR98 [43]. USH2A, GPR98, WHRN and PDZD7 interact to assemble a multiprotein complex at the ankle link region of the mechanosensitive stereociliary bundle in hair cells. Defects of any proteins in this complex should cause stereociliary bundle disorganization and deafness. From this analysis, we suggested that the mutated GPR98 domain has drastically decreased protein’s functionality and complex stability due to the partial unfolding of the 7TM-GPCRs domain. Hopefully, NGS technique with the molecular simulation trajectory will be guidance in revealing the potential phenotype-genotype correlation, and diagnosis and treatment of this disease [44].

The offspring of consanguineous marriages are at higher risk of genetic disorders. Autosomal recessive disorders carry two copies or alleles of the same *GPR98* gene mutation in the consanguineous marriage, one of each inherited from their grandparents. Both parents of an individual with this USH2C are carriers of *GPR98* gene, displaying no signs of diseases, and might not be aware that they carry the same mutated gene. However, their children are at a 25% risk in those autosomal recessive disorder families. In this regard, it is very important to provide genetic counseling and family planning for the consanguineous marriage pedigree in un-developed and developing countries, including Tunisia and China.

## Conclusions

Our findings showed the possibility that the novel homozygous p.Leu2305Valfs*4 frameshift variant of GPR98 is deleterious and disease-causing, according to the NGS-based comprehensive genetic evaluation of a USH2C variation in a consanguineous marriage Chinese family. These results may help our understanding of the variant that contributes to the susceptibility or causativity of USH progression. Thus, our study indicates that p.Leu2305Valfs*4 variant could be novel and pathogenic, that risks for carriers and their potential offspring must be informed to USH2C patients and their families. Genetic counseling and family plan for consanguineous marriage pedigree is also necessary.

## Abbreviations

FA: Fluorescein angiogram
GPR98: G protein-coupled receptor 98
NGS: Next generation sequencing
OCT: Optical coherence tomography
USH2C: Usher syndrome type IIC
